# Blended Working: For Whom It May (Not) Work

**DOI:** 10.1371/journal.pone.0102921

**Published:** 2014-07-17

**Authors:** Nico W. Van Yperen, Eric F. Rietzschel, Kiki M. M. De Jonge

**Affiliations:** Department of Psychology, University of Groningen, Groningen, The Netherlands; University of Leicester, United Kingdom

## Abstract

Similarly to related developments such as *blended learning* and *blended care*, *blended working* is a pervasive and booming trend in modern societies. Blended working combines on-site and off-site working in an optimal way to improve workers’ and organizations’ outcomes. In this paper, we examine the degree to which workers feel that the two defining features of blended working (i.e., time-independent working and location-independent working) enhance their own functioning in their jobs. Blended working, enabled through the continuing advance and improvement of high-tech ICT software, devices, and infrastructure, may be considered beneficial for workers’ perceived effectiveness because it increases their job autonomy. However, because blended working may have downsides as well, it is important to know *for whom* blended working may (not) work. As hypothesized, in a sample of 348 workers (51.7% women), representing a wide range of occupations and organizations, we found that the perceived personal effectiveness of blended working was contingent upon workers’ psychological need strength. Specifically, the perceived effectiveness of both time-independent working and location-independent working was positively related to individuals’ need for autonomy at work, and negatively related to their need for relatedness and need for structure at work.

## Introduction

Technological revolutions and the widespread adoption of Information and Communication Technologies (ICTs) have shifted our notions about the location, purpose, and value of our work [1]. The rapid advance of ever improving ICTs in modern societies enables smooth and seamless *time-independent working* (i.e., flexibility in *when* and *how long* workers engage in work-related tasks) and *location-independent working* (i.e., flexibility in *where* work gets done), henceforth labeled as *blended working*. Blended working combines on-site and off-site working (including online or e-working) in an optimal way to improve workers’ and organizations’ positive outcomes (e.g., productivity, satisfaction, motivation, collaboration, and workplace utilization), and to reduce negative outcomes (e.g., absenteeism, tardiness, turnover, and time loss). In this paper, we examine the degree to which workers feel that the two defining features of blended working (i.e., time-independent working and location-independent working) enhance their own functioning in their jobs. In addition, we address the question whether this perceived effectiveness depends on individual need strength. For example, blended working may generally be considered beneficial because it increases workers’ job autonomy, but for some workers, this may actually create ambiguity [2]. Hence, in this paper we argue and demonstrate that the perceived effectiveness of blended working is contingent upon workers’ psychological need strength.

### Blended working in the modern workplace

Through the rise of ICTs such as the internet, e-mail, video calling, chat, and cloud-based computing, the world of work has changed in unprecedented ways. Information has become a commodity and the key driver in many industries and occupations. One of the consequences of this development is a tremendous increase over the last 30 years or so in the number of information or knowledge workers who have the ability and opportunity to decide when, where, and for how long to engage in work-related tasks [1], [3]. More and more often, to collaborate effectively, workers do not need to be in the same location or area, and do not have to be working in the same time zone. If real-time contact is needed, this can often take place through ICTs. Moreover, because it has become less important where and when people perform their jobs, workers have the possibility of scheduling their work tasks in the ways that best suit them, rather than having to follow traditional office hours.

In this paper, we use the term *blended working* to refer to time-independent and location-independent working enabled through high-tech ICT software, devices, and infrastructure. Similarly to related pervasive and booming trends in modern societies, including *blended learning* [4] and *blended care* [5], blended working creates opportunities for improving individuals’ and organizations’ desired outcomes in ways that were not previously possible [6], [7]. This trend will continue to grow because of, among other things, the continuing advance and improvement of ICT software, devices (computers, tablets, hand-held and wearable devices, etc.), and infrastructure (i.e., increased availability of high-speed broadband connections). For an increasing number of tech-savvy workers who wish to build flexibility into their work and non-work lives, this development gives them unprecedented control of location, time, and pace [8].

Obviously, blended working is not for everyone: certain jobs can only be done on-site, such as work in plants, hospitals, schools, and courts. However, research indicates that 45% of the US workforce (excluding self-employed workers) utilizes–at least part-time–a technology-centric flexible work environment, whether they are at the traditional office, travelling, or at home [8]. These workers (primarily) create, analyze, communicate, and use digital information in performing their jobs. Figures show that the number of US workers (either working for an employer or self-employed) who have worked from home or remotely for an entire day at least once a month since 2005 is more than 25 million, representing nearly 20% of the US working adult population [9], [12]. Approximately 50% of the US workers who exclusively worked from a traditional office indicated that they were (very) interested in working from home, and only 20% demonstrated no interest at all [1], [8].

### The possible consequences of blended working

Because of this rapidly growing interest in blended working, the consequences of work flexibility for work-life balance, psychological health, and work performance are becoming increasingly relevant and important. Through increased flexibility and a better work-home balance, blended working may have substantial positive consequences for workers’ effectiveness and quality of work and life [1], [10]–[14]. In addition, blended working is a way for companies to reduce expenses, including real estate costs [15], to comply with government regulations regarding equal opportunities, and to demonstrate corporate social responsibility [16]. For example, due to reduced office space requirements, traffic, paper use, etc., blended working may have positive consequences for the environment, such as the reduction to sustainable levels of carbon dioxide emissions, deforestation, and greenhouse gases, and less need for additional office space and highway capacity [1], [8].

Blended working may have downsides as well, particularly for those working primarily away from the office. These include relational and information impoverishment at work, ambiguity about tasks and roles, career stagnation (“out of sight, out of mind”), increased work-home interference, distraction and interruption by family members (particularly when there is no detached home office space), procrastination, cyberslacking, and the pressure to be available anywhere, at any time [13], [17]–[19]. However, a large-scale meta-analysis indicated that work arrangements allowing workers to perform their tasks while being remote from their office had mainly beneficial effects on work-home balance, job satisfaction, and job performance, and generally had no detrimental effects on the quality of relationships at work or on perceived career prospects [11]. Importantly, however, this meta-analysis also showed that the effects of these work arrangements were moderated by variables such as the intensity of teleworking, experience with teleworking, and sex. Thus, the consequences of blended working may be more favorable (or unfavorable) for some people than for others, which raises the question *for whom* blended working is likely to work [6], [20].

The availability of high-tech ICTs is clearly the baseline needed to implement, maintain, and improve blended working, but its success will ultimately be a function of the degree to which workers feel that time-independent working and location-independent working enhance their own functioning and productivity in their jobs. In the present study, we argue and demonstrate that this *perceived personal effectiveness* of blended working, in turn, depends on the strength of workers’ psychological needs, that is, their need for autonomy, need for relatedness, need for competence, and need for structure.

### The defining features of blended working and psychological needs strength

The two defining features of blended working are *time-independent working* and *location-independent working*. Regarding the former, workers can, for example, choose to maintain traditional office hours, or to work in the evening, on weekends, or any combination of these. Regarding location-independent working, workers may work at the office (which can include ‘hot desking’), at home, while traveling, or at ‘neutral’ workplaces that are shared, swapped, reserved, rented, or simply claimed for a time, etc. In the current study, workers’ perceived effectiveness of both time-independent and location-independent working was expected to be a function of four important psychological needs: the needs for autonomy, relatedness, competence, and structure [21], [22]. In line with typical use in organizational theories, these needs were treated as individual difference variables, which means that workers were considered as differing in need strength [21]. Hence, workers who have a strong dispositional need for autonomy may feel that blended working works for them, because they get more discretion as to where and when to work. In contrast, workers with a high need for a structured and unambiguous environment may perceive blended working as personally ineffective because it fuels their aversion to ambiguity. In other words, perceived effectiveness is likely to depend on the degree to which blended working fits workers’ specific psychological needs.

**Need for autonomy** is defined as individuals’ desire to feel volitional and to experience a sense of choice and psychological freedom [23], [24]. Blended working obviously increases workers’ job autonomy through time- and location-independent working, which typically has favorable effects on work and organizational outcomes [25], [26]. Hence, we hypothesized (*Hypothesis 1*) that workers’ need for autonomy *positively* predicts the perceived effectiveness of blended working (i.e., time-independent working and location-independent working).

**Need for relatedness** refers to individuals’ wish to feel connected to others and to be a member of a group [24], [27]. Blended working entails working away from the office and having flexible working hours, which may cause workers to feel disconnected from others and socially isolated [18], [28]. This obviously would interfere with workers’ need for relatedness. Accordingly, we hypothesized (*Hypothesis 2*) that workers’ need for relatedness *negatively* predicts the perceived effectiveness of blended working.

**Need for competence** is defined as individuals’ desire to feel competent and skilled [23]. Need for competence is positively associated with the wish to engage in challenging tasks, to acquire new skills, and to perform well [24]. Blended working can be considered a challenging, innovative work arrangement which likely satisfies workers’ need for competence. Accordingly, we hypothesized (*Hypothesis 3*) that workers’ need for competence *positively* predicts the perceived effectiveness of blended working.

**Need for structure** refers to a strong preference for structure and predictability and a low tolerance for ambiguity [22]. Need for structure, or intolerance for ambiguity, is positively related to feedback-seeking behaviors [29] and a preference for managers who are inclined to guide their subordinates by planning and scheduling work tasks [30]. In line with these findings, workers high in need for structure may dislike time and location-independent working because it tends to create ambiguity [2]. Hence, we hypothesized (*Hypothesis 4*) that workers’ need for structure *negatively* predicts the perceived effectiveness of blended working.

## Method

### Ethics Statement

This research was approved by the ethics committee of the Department of Psychology, University of Groningen. The data were analyzed anonymously.

### Sample and Procedure

The sample consisted of 348 workers (51.7% women), representing a wide range of industries, including Public Administration (32.5%), Banking, Insurance, and Business Consultancy (20.1%), Information Technology (9.2%), Education (6.6%), Healthcare and Social Assistance (4.6%), Research and Science (3.7%), and Communication and Media (3.4%). Type of employment was permanent (83.3%), temporary (10.3%), self-employed (5.5%), or ‘other’ (.9%); 23.6% had a management position. The mean age was 44.45 years (*SD* = 9.96), and 67.7% had one or more children (including adopted children and stepchildren) living at home. The average number of self-reported working hours per week was 38.11 hours (*SD* = 8.16). Educational level varied from senior general education or secondary vocational education (13.5%) to university education at bachelor’s level (50.0%) or MSc/PhD level (36.5%).

Workers were approached through an announcement posted on a social networking website for people in professional occupations, or in (digital) letters or email messages in a variety of companies. The participants followed the link to the questionnaire, which was voluntarily completed over the internet. Only respondents that indicated working at least 8 hours a week were included. Furthermore, to safeguard data quality, we used instructed response items to filter out invalid responses, as recommended by Mason and Suri [31].

### Measures

#### Perceived personal effectiveness of blended working

This new measure comprises two three-item subscales (see Table 1) that represent the core features of blended working, that is, time-independent working and location-independent working.

**Table 1:** Specified Three-Factor Solution of a Principal-Components Analysis of the Two Defining Features of Blended Working and Work-home Segmentation Preference.

1 = Strongly disagree			
2 = Mainly disagree			
3 = Slightly disagree			
4 = Neither agree nor disagree			
5 = Slightly agree			
6 = Mainly agree			
7 = Strongly agree			
**Please indicate to what extent you (dis)agree with each of these statements.**There are no “right” or “wrong” answers. We are only interested in what **you** think.			
	**1**	**2**	**3**
**Time-independent working**			
1. I function best when my working hours are flexible.			.860
2. Flexible working hours enhance my own productivity.			.784
3. I can work effectively at almost any time.			.761
**Location-independent working**			
4. I can do my job well at several locations.		.944	
5. Also if I am not working at the office (but elsewhere), I can be very productive.		.864	
6. From any working place I can maintain my work relationships with colleagues, executives, customers, etc.		.525	
**Work-home segmentation preference**			
7. I don’t like work issues creeping into my home life.	−.876		
8. I prefer to keep work life at work.	−.840		
9. I don’t like to have to think about work while I’m at home.	−.813		
10. I like to be able to leave work behind when I go home.	−.787		
**Eigenvalue**	4.428	1.750	.896
**% of Variance**	44.28	17.50	8.96

**Note:** Factor loadings higher than |.50| on the rotated factors (oblimin rotation with Kaiser normalization) are presented.

**Work-home segmentation preference** was assessed by using the four-item scale developed by Kreiner [32], which measures the degree to which workers prefer a workplace that helps segment work and home domains (for the items, see Table 1).

The 10 statements representing the three subscales (i.e., time-independent working, location-independent working, and work-home segmentation) were subjected to a rigorous psychometric examination. As shown in Table 1, the pattern matrix (principal component analysis) demonstrate that the three subscales represent both conceptually and empirically distinct concepts.

#### Psychological need strength

Drawing on existing measures used to assess need satisfaction (autonomy, relatedness, and competence) rather than need strength [24] and Personal Need for Structure [22], we developed four short, equally formatted psychological need strength measures for the strength of workers’ need for autonomy, need for relatedness, need for competence, and need for structure. The advantage of equally formatted need strengths scales is that the strengths of the different needs can be compared with each other, which is interesting in itself (see Results section). The 16 items representing the four psychological needs at work were subjected to a principal component analysis. The results show that the four psychological needs are both conceptually and empirically distinct concepts (see Table 2).

**Table 2:** Specified Four-Factor Solution of a Principal-Components Analysis of the Need Strength Measures.

1 = not at all				
2 = to a very small extent				
3 = to a small extent				
4 = to a moderate extent				
5 = to a large extent				
6 = to a very large extent				
7 = to an extremely large extent				
**At work** I have the need …				
	**1**	**2**	**3**	**4**
**Need for autonomy**				
1. …. to decide on my own how to go about getting my job done.				.882
2. …. to have a say in determining my activities and tasks.				.866
3. …. to determine on my own how to best approach my work.				.855
4. …. for freedom to do my work in the way that I think is best.				.783
**Need for relatedness**				
5. …. to hang out with people.			−.929	
6. …. to be with other people.			−.900	
7. …. to be around people so I do not feel alone.			−.758	
8. …. to feel like I am part of a team or a group.			−.726	
**Need for competence**				
9. …. to feel that I have the knowledge and skills to do my work well.		.860		
10. …. to feel skilled.		.841		
11. …. to feel that I can finish difficult tasks successfully.		.798		
12. …. to be good at my work.		.770		
**Need for structure**				
13. …. for a daily routine.	.864			
14. …. for order and regularity.	.843			
15. …. to know exactly what to expect.	.801			
16. …. for rules and guidelines that I can follow.	.769			
**Eigenvalue**	4.256	3.532	1.953	1.591
**% of Variance**	26.60	22.07	12.21	9.94

**Note:** Factor loadings higher than |.50| on the rotated factors (oblimin rotation with Kaiser normalization) are presented.

## Results

### Descriptive data

The means, standard deviations, correlations, and Chronbach’s alphas are presented in Table 3. As expected, workers with a stronger work-home segmentation preference perceived both features of blended working as less personally effective, which provides empirical support for the construct validity of the new measure of blended working.

**Table 3:** Means, Standard Deviations, Correlations, and Cronbach’s Alphas (n = 348).

	*M*	SD	1	2	3	4	5	6	7	8	9	10	11
Age	44.45	9.96	−										
#Children at home	1.11	1.01	.23	-									
Educational Level	2.23	.67	−.18	−.03	-								
#working hours	38.11	8.16	.05	−.05	.25	-							
Need for Autonomy	5.52	.71	−.01	−.06	.25	.21	**.88**						
Need for Relatedness	3.76	.99	−.20	−.08	.05	.03	−.14	**.85**					
Need for Competence	5.30	.69	−.07	−.05	.02	.02	.26	.20	**.84**				
Need for Structure	2.90	1.00	−.18	−.12	−.24	−.16	−.32	.28	.07	**.85**			
Time-independent working	5.05	1.24	.05	.15	.05	.10	.29	−.22	.04	−.30	**.78**		
Location−independent working	5.77	1.04	.02	.03	.04	.07	.31	−.23	.07	−.21	.52	**.73**	
Segmentation Preference	3.80	1.38	−.14	−.14	−.09	−.13	−.29	.16	−.07	.38	−.52	−.27	**.87**

**Notes:** Pearson correlations higher than. 13 are significant at the .01 level. Cronbach’s Alphas are presented on the diagonal.

The negative correlations with age indicate that older workers were lower in need for relatedness and need for structure, and they had a weaker preference for work-home segmentation. Having more children at home was associated with a stronger preference for time-independent working and a weaker preference for work-home segmentation. Furthermore, educational level and number of working hours were related to a higher need for autonomy and a lower need for structure.

The links between the unrelated (χ_(1)_^2^ = 1.25, *p* = .31) dichotomous variables leadership position and sex, on the one hand, and the 11 variables presented in Table 3, on the other, were examined using a Multivariate Analysis of Variance (MANOVA). Overall, there were differences between leaders and non-leaders, *F*(11, 336) = 2.33, *p* = .009. Relative to non-leaders (*p*s<.01), leaders worked more hours per week (*M*_l_ = 40.90, *SD* = 7.76 vs. *M*_non-l_ = 37.25, *SD* = 8.10), were higher in need for autonomy (*M*_l_ = 5.73, *SD* = .63 vs. *M*_non-l_ = 5.45, *SD* = .73), lower in need for structure (*M*_l_ = 2.65, *SD* = 1.02 vs. *M*_non-l_ = 2.97, *SD* = .98), and they had a weaker preference for work-home segmentation (*M*_l_ = 3.47, *SD* = 1.32 vs. *M*_non-l_ = 3.90, *SD* = 1.38). The significant sex differences (*F*(11, 336)  = 7.45, *p*<.001) indicated that relative to women (*p*s<.01), men were older (*M*_men_ = 47.16, *SD* = 9.37 vs. *M*_women_ = 41.93, *SD* = 9.85) and worked more hours per week (*M*_men_ = 40.86, *SD* = 7.30 vs. *M*_women_ = 35.55, *SD* = 8.09).

We next compared the strengths of the different psychological needs at work with each other. Pairwise comparisons revealed that the differences between the need strength subscales were significant at the.001 level (*t*-values>4.61). As shown in Table 3, workers’ strongest psychological need was their need for autonomy, followed by their need for competence, their need for relatedness, and their need for structure.

### Hypothesis testing

To examine which psychological needs at work predicted the perceived effectiveness of each feature of blended working as well as work-home segmentation preference, three separate regression analyses were conducted with time-independent working, location-independent working, and work-home segmentation preference, respectively, as the dependent variables, and the four different psychological needs as the predictor variables.

Table 4 (*cf.,* Table 3) shows that we found empirical support for *Hypothesis 1*: workers’ need for autonomy positively predicts their perceived effectiveness of blended working (i.e., time-independent working and location-independent working). These findings are in line with the negative link between need for autonomy and work-home segmentation preference (see Tables 3 and 4). Furthermore, the higher workers’ need for relatedness at work, the lower their perceived effectiveness of time-independent working and location-independent working, which provides support for *Hypothesis 2*.

**Table 4:** Results of Regression Analyses for the Features of Blended Working (and Work-Home Segmentation Preference) on the Control Variables and Predictor Variables (n = 348).

	Workers’ perceived effectiveness of blended working								
	Time-independent working			Location-independent working			Work-Home Segmentation Preference		
Step 1: Control variables									
	b	β	t	b	β	t	b	β	t
1. Age	−.01	−.07	−1.18	−.01	−.07	−1.21	−.01	−.05	−.97
2. #Children at home	.18	.14	2.77**	.02	.02	.41	−.14	−.11	−2.08*
3. Educational Level	−.12	−.06	−1.14	−.07	−.04	−.78	.03	.02	.30
4. #working hours	.01	.05	.93	−.001	−.01	−.19	−.01	−.06	−.99
5. Leadership position	-.02	−.02	−.29	−.04	−.03	−.58	−.09	−.06	−1.09
6. Sex	.02	.02	.33	.10	.10	1.71	.03	.02	.39
*F*(6,341)	2.10*			.58			4.16***		
R^2^	.04*			.01			.07***		
**Step 2: Predictor variables**									
	**b**	**β**	**t**	**b**	**β**	**t**	**b**	**β**	**t**
7. Need for Autonomy	.38	.22	3.84***	.37	.25	4.34***	−.31	−.16	−2.85**
8. Need for Relatedness	−.18	−.14	−2.60**	−.19	−.18	−3.36**	.08	.06	1.04
9. Need for Competence	.05	.03	.53	.09	.06	1.04	−.12	−.06	−1.17
10. Need for Structure	−.24	−.19	−3.37**	−.11	−.11	−1.88	.40	.29	5.12***
*F*(4,337)	14.07***			14.09***			14.10***		
R^2^_change_	.14***			.14***			.13***		

**Notes:** Sex (+1 men, −1 women) and Leadership Position (+1 leadership position, −1 no leadership position) were dummy coded. The regression weights concern the analysis in which all effects were entered: **b** = the unstandardized regression weight, **β** = the standardized regression weight, **t** = t-value.

*p<.05.

**p<.01.

***p<.001.

We also found support for the hypothesis (*Hypothesis 4*) that workers’ need for structure at work negatively predicts their perceived effectiveness of blended working (see Table 3), although in the regression analyses (see Table 4), the link between need for structure and the perceived effectiveness of location-independent working was only marginally significant (*p* = .06). Need for structure was also positively related with workers’ preference for work-home segmentation (see Tables 3 and 4). The only unexpected result was the nonsignificant link between workers’ need for competence at work and their perceived effectiveness of blended working (including their preference for work-home segmentation, see Tables 3 and 4), so that no empirical evidence was obtained for *Hypothesis 3*.

## Discussion

In the present paper, we introduce and operationalize the concept of blended working, that is, smooth and seamless work flexibility enabled through high-tech ICTs. Blended working combines on-site and off-site working in an optimal way to improve workers’ and organizations’ positive outcomes and to reduce negative outcomes. However, blended working may also have unfavorable consequences for workers and organizations [1], [10]–[11], [13], [14], [15], [19], [33]. Therefore, it is important to know *for whom* blended working may (not) work. In this first study on blended working, we argued and demonstrated that the degree to which workers perceived the two defining features of blended working (time-independent working and location-independent working) to contribute to their personal effectiveness at work, was a function of their psychological need strengths. Specifically, the stronger their need for autonomy at work, and the weaker their needs for relatedness and structure at work, the more workers felt that blended working was effective for them.

The contribution of this study is twofold. Firstly, we introduced and operationalized the concept of blended working and, accordingly, presented a manageable measure of workers’ perceived personal effectiveness of blended working. We found that workers’ perceived personal effectiveness of time-independent working, location-independent working, and work-home segmentation preference, were both conceptually and empirically distinct. Furthermore, workers with a stronger work-home segmentation preference perceived both features of blended working as less personally effective, which provides empirical support for the construct validity of the new measure of the perceived personal effectiveness of blended working. Similarly, the four short, equally formatted psychological need strength measures (i.e., needs for autonomy, relatedness, competence, and structure at work) appeared to be both conceptually and empirically distinct. In future research, these newly developed measures may be useful for researchers interested in blended working or psychological need strength.

Secondly, because the consequences of blended working may be more favorable (or unfavorable) for some people than for others, we addressed the question *for whom* blended working is likely to work. More specifically, we examined which psychological needs at work explain workers’ perceived personal effectiveness of time-independent working and location-independent working. New work arrangements such as blended working will be successful only if they fit workers’ psychological needs. Because blended working increases workers’ job autonomy through time- and location-independent working, particularly workers high in need for autonomy perceive blended working as highly personally effective. This, in turn, may have a positive effect on important work and organizational outcomes [25], [26]. However, blended working can also entail working away from the office and having flexible working hours, which could cause workers to feel socially isolated [18], [28]. In addition, workers high in need for structure may dislike time and location-independent working because it tends to create ambiguity [2]. Indeed, our findings suggest that blended working is most suitable for workers who are high in need for autonomy, low in need for relatedness, and low in need for structure.

Rather than suggesting a general profile of the ‘ideal blended worker,’ our results show that the perceived effectiveness of the core features of blended working is contingent upon workers’ psychological needs strength. Similarly, research on Person-Environment (P-E) fit [34] indicates that workers are most motivated and perform best when the requirements and affordances of the (physical and non-physical) work environment are aligned with their own needs and abilities. Thus, the implementation of blended working practices should not be seen as an ‘all or nothing’ or ‘one size fits all’ issue. Rather, to find the right solution for every employee, each individual’s work-related psychological needs strengths should be considered to assess how well it fits with the core features of blended working.

We did not find empirical evidence for our expectation that need for competence at work would be positively related to workers’ perceived effectiveness of blended working. Possibly, workers engaged in blended working practices may perceive this way of working as challenging and innovative only for a short period of time. Moreover, blended working practices may not satisfy workers’ need for competence because perceiving a job as new and challenging may be primarily a function of work content rather than work arrangement. Future work might attempt to disentangle the different roles of work content and work engagement and the ways in which these interact with different psychological needs.

### Demographic variables

The observed links between demographic variables and the perceived personal effectiveness of blended working were weak or non-existent. Only number of children was positively related to the perceived effectiveness of time-independent working. Indeed, particularly for parents, blended working may create opportunities for combining working life and childcare that were not previously available, which are likely to improve and maintain their work-life balance [35]. However, this link was not very strong (*r* = .15, see Table 3), and there was no relationship with location-independent working, which suggests that blended working may be attractive not only for parents.

Although age was not related to the perceived effectiveness of blended working, significant, negative relationships were observed with work-home segmentation preference, need for structure, and need for relatedness (see Table 3), which may suggest that workers perceive blended working as more personally effective as they are older rather than younger. Indeed, others found that age is positively correlated with the tendency to work from home [8]. Younger workers may be less likely to work from home because they are eager to learn from more experienced colleagues, and because they may feel that working from home negatively impacts their chances of advancement. Older workers may thrive on their expertise and experience, and have had the time to earn the trust that is essential to working remotely and to self-managing their work. However, an opposing tendency may be that e-working is more suited to younger, more tech-savvy workers who grew up with technology, which may explain the absent correlations between age and the perceived effectiveness of blended working. Thus, it might be the case that attitudes towards blended working are mediated by different psychological mechanisms for different age groups; this might be an interesting avenue for future research.

### Future research and conclusion

We identify several possible lines of future research, in addition to those briefly alluded to above. Firstly, the current study focused on workers’ *perceptions* of blended working effectiveness. While this is an important first step, what counts in the end is actual effectivenes, i.e., the degree to which blended working contributes to (or hinders) workers’ performance and wellbeing. Future research might therefore focus on the effects of blended working on actual changes in workers’ effectiveness and productivity, rather than their own perceived effectiveness. Furthermore, it would be interesting to study additional individual difference variables that may be beneficial or detrimental to the perceived and actual effectiveness of blended working, including conscientiousness, procrastination, and type of achievement motivation [36]. If similar patterns are consistently observed across different samples, the results can be used to optimize selection procedures for blended workers. Finally, future research might also focus on contextual variables that may facilitate the effectiveness of blended working [37]. Possible candidates are leadership style (controlling vs. empowering), feedback style (process vs. output oriented), and social norms (favorable vs. unfavorable). These results could then be used to develop effective interventions and best practices of blended working. Ideally, future studies would also employ multi-source and multilevel methods, for example, by collecting data on blended working practices at the organizational level (e.g., the degree to which organizations offer possibilities for blended working), and by having supervisors rate workers’ effectiveness.

In conclusion, we hope that this first study on blended working will serve as a catalyst for future research aiming at a better understanding of the effectiveness of blended working practices. It seems clear that blended working is not for everybody. Given the rapid development and widespread adoption of blended working practices, a deeper understanding of the contingencies surrounding blended working effectiveness is clearly needed.
